# Graft Transmission of RNA Silencing to Non-Transgenic Scions for Conferring Virus Resistance in Tobacco

**DOI:** 10.1371/journal.pone.0063257

**Published:** 2013-05-22

**Authors:** Emran Md. Ali, Kappei Kobayashi, Naoto Yamaoka, Masayuki Ishikawa, Masamichi Nishiguchi

**Affiliations:** 1 Faculty of Agriculture, Ehime University, 3-5-7 Tarumi, Matsuyama, Japan; 2 Plant-Microbe Interactions Research Unit, National Institute of Agrobiological Sciences, 2-1-2 Kan-nondai, Tsukuba, Ibaraki, Japan; Swedish University of Agricultural Sciences, Sweden

## Abstract

RNA silencing is a mechanism of gene regulation by sequence specific RNA degradation and is involved in controlling endogenous gene expression and defense against invasive nucleic acids such as viruses. RNA silencing has been proven to be transmitted between scions and rootstocks through grafting, mostly using transgenic plants. It has been reported that RNA silencing of tobacco endogenous genes, *NtTOM1* and *NtTOM3*, that are required for tobamovirus multiplication, resulted in high resistance against several tobamoviruses. In the present study, we examined the graft transmission of RNA silencing for conferring virus resistance to non-transgenic scions of the same and different *Nicotiana* species grafted onto rootstocks in which both *NtTOM1* and *NtTOM3* were silenced. Non-transgenic *Nicotiana tabacum* (cvs. Samsun and Xanthi nc) and *N. benthamiana* were used as scions for grafting onto the rootstocks silenced with both genes. Short interfering RNA (siRNA) of *NtTOM1* and *NtTOM3* was detected in both the scions and the rootstocks eight weeks after grafting. The leaves were detached from the scions and inoculated with several tobamoviruses. The virus accumulation was tested by ELISA and northern blot analysis. The viruses were detected in grafted scions at extremely low levels, showing that virus resistance was conferred. These results suggest that RNA silencing was induced in and virus resistance was conferred to the non-transgenic scions by grafting onto silenced rootstocks. The effect of low temperature on siRNA accumulation and virus resistance was not significantly observed in the scions.

## Introduction

RNA silencing is sequence-specific gene regulation, which is widely conserved among eukaryotes including fungi, plants and animals. One of the characteristics is a mobile signal that can spread to neighboring cells through plasmodesmata or systemically to the entire plant through the vascular system [1]–[4]. Long distance or systemic silencing involves an amplification of the same silencing signal utilizing cellular RNA-dependent RNA polymerase (RDR) [4], [5]. Double-stranded (ds) RNA is the key trigger for RNA silencing. There are different ways for generating dsRNA: as partial ds structure of viral RNA, by which they can induce RNA silencing and become targets for RNA degradation [6], transcription of inverted repeats (IR) [7], [8] or RDR-mediated synthesis that uses aberrant RNA (aRNA) as a template [9]. Dicer, a ribonuclease (RNase) III family, is involved in the cleavage of the dsRNA into 21–25 nucleotide (nt) duplexes, referred to as short interfering RNA (siRNA) [10], [11]. The next step in the RNA silencing process is the incorporation of siRNA into Argonaute containing (Ago-containing) multicomponent RNA-induced silencing complex (RISC). After an ATP-dependent incorporation of (−) siRNA into RISC, the Ago acts as a sequence specific ribonuclease (Slicer) that cleaves single stranded RNA at discrete positions [11]–[13].

*Arabidopsis thaliana* genes *TOM1* and *TOM3* are involved in tobamovirus multiplication [14]–[16]. In the double null mutants of these genes, tobamovirus cannot multiply [15]. These genes are conserved in other plants including tomato, tobacco and melon [17]. It is reported that *N. tabacum* carries two endogenous homologous genes *NtTOM1* and *NtTOM3* which function in parallel to support tobamovirus multiplication and that silencing of both genes resulted in high resistance against several tobamoviruses [17].

It is widely accepted that the siRNA signal in plants can spread systemically. The siRNA signal moves through sieve tubes and serves an antiviral function [18]. Recently, siRNA was shown to be able to pass through the graft junction and that siRNA triggers RNA silencing in recipient cells [19], [20]. Most experiments on the graft transmissions of RNA silencing have been shown with transgenic plants [1], [21]–[25]. The transmission of post-transcriptional gene silencing (PTGS) to transgenic scions occurred from transgenic tobacco plants silenced with both the transgene and endogenous gene of the nitrate reductase gene (Nia) but that PTGS was never transmitted to non-transgenic scions [1]. More recently it is reported that silencing of endogenous genes in non-transgenic scions was induced from transgenic rootstocks in which the companion cell specific promoter driven hairpin construct (hp) of the glutamate-1-semialdehyde aminotransferase (GSA) gene was introduced by *Agrobacterium* but not from silenced rootstocks with cauliflower mosaic virus (CaMV) 35S promoter-driven hpGSA [26]. Another report on grafted apple showed that graft transmission of RNA silencing to *GUS* expressing scions occurred in an *in vitro* system from *GUS* silenced transgenic apple but that it did not to non-transgenic scions from rootstocks silenced with an endogenous *Malus domestica* anthocyanindin synthase (*Mdans*) gene [27]. In this study, we show that RNA silencing and the resultant virus resistance was induced in non-transgenic scions through grafting onto transgenic silenced rootstocks, where CaMV 35S promoter-driven hp construct of the endogenous gene of tobacco *NtTOM1* and *NtTOM3* were silenced. Our findings may shed light on a practical approach of RNA silencing to confer desired traits to non-transgenic plants.

## Materials and Methods

### Plant Materials

Transgenic Sd1 (Silencing double mutant 1) line, in which both *NtTOM1* and *NtTOM3* genes were silenced, was produced by crossing transgenic S-tom1 and S-tom3 plants [17]. The Sd1 line was selected after selection by kanamycin. Non-transgenic *Nicotiana tabacum* (cvs. Samsun and Xanthi nc) and *N. benthamiana* were used as scions for grafting onto the Sd1 rootstocks. Seeds were sown on ½ MS plates and one week later the seedlings were transferred to plastic plant boxes containing ½ MS with kanamycin (50 µg/ml). Approximately 2 weeks later the plants were transferred to pots containing autoclaved soil (Fujimi Co. Ltd., Shizuoka, Japan) in growth chambers. The plants were grown at 24°C under a 16-light/8-h dark cycle with cool fluorescent light.

### Grafting Procedure

Approximately 8-week-old plants were used for the cleft grafting, as described previously [21]. Rootstocks were prepared by removing the shoots above at least two basal leaves and then creating a vertical cut of 1–2 cm at the center of the stem. Scions (3–5 cm stature) were prepared by removing leaves and trimming the base of the scion to a wedge. The scion/rootstock junction was wrapped with Parafilm and a clip. Plants were covered with plastic bags to avoid dehydration for 1 week or until the graft was completed.

### DNA Extraction and PCR Analysis

DNA was extracted from leaves using a cetyltrimethylammonium bromide-based (CTAB-based) extraction procedure [28]. Quantity and purity of DNA was measured by using a spectrophotometer (Gene Spec I, Hitachi Co. Ltd., Tokyo, Japan). Genomic DNA (100 ng) was used as a template for PCR. The forward primer GUS-linker-F and the reverse primers NtTOM1-R or NtTOM3-R were used to amplify the *NtTOM1* or *NtTOM3* transgene, respectively. The PCR reaction was 2 min of 94°C pre-heating, followed by a 25-cycle amplification program; 1 min at 94°C for denaturation, 1 min at 55°C for annealing and 1 min at 72°C for extension, and a final extension at 72°C for 5 min. PCR products were analyzed by electrophoresis on a 1% agarose gel. Forward and reverse primer pairs are listed in Table S1.

### RNA Isolation and Reverse Transcription PCR

Total RNA was extracted from the young leaves of the transgenic Sd1 line and non-transgenic plants using a TRI reagents kit (Molecular Research Centre, Cincinnati, OH, USA) according to the manufacturer's instructions, and residual genomic DNA was removed by DNase I treatment for 30 min at 37°C (Takara, Kyoto, Japan). Total RNA (1 µg) was reverse-transcribed using RevertAid^TM^ reverse transcriptase (Fermentas, Hanover, CA, USA) in a 20 µl reaction containing 5X RT buffer [250 mM Tris-HCl (pH 8.3 at 25°C), 250 mM KCl, 20 mM MgCl_2_, 50 mM DTT], 10 mM dNTP, 20U RNase Inhibitor and 20 µM of GUS-linker-R, NtTOM1-R or NtTOM3-R primer. The mixture containing RNA and primers was heated at 70°C (10 min) and chilled (10 min) before addition of 5X buffer and RNase inhibitor, and then incubated at 42°C (60 min). The reaction was inactivated at 70°C (10 min). The cDNA was amplified by PCR under the following conditions: 95°C for 1 min, followed by 30 cycles of denaturing at 95°C for 30 sec, annealing at 60°C for 30 sec, and extension at 68°C for 1 min, and a final extension at 68°C for 10 min. The forward primer NtTOM1-F or NtTOM3-F and the reverse primer NtTOM1-R, NtTOM3-R or GUS-linker-R were used to amplify the endogenous/transgene *NtTOM1 or NtTOM3,* respectively. Their sequences are listed in Table S1. PCR products were analyzed by electrophoresis on a 1% agarose gel.

### Probe DNA Preparation

The cDNA clones, pBI-NtTOM1 and pBI-NtTOM3, were digested with the combination of *Xba*I/*Sac*I and *Bam*HI/*Sac*I, respectively. The gel-purified DNA fragments (876 and 910 bp) of pBI-NtTOM1 and pBI-NtTOM3 were used as *NtTOM1* and *NtTOM3* probes, respectively. To detect virus accumulation, the 600 bp *Eco*RI/*Bam*HI fragment of pTMVOM-L (Kawamura and Ishikawa, unpublished), 1964 bp *Kpn*I/*Eco*RI fragment of pL11A-2-35 [29], and 900 bp *Eco*RI DNA fragment of a WMoV cDNA clone, pTW62-2 [30], were used as probes to detect the accumulation of TMV, ToMV and WMoV, respectively. The GUS fragment from pBI221 was prepared after digestion with *Bam*HI/*Sac*I. The [*α*-^32^P]dCTP-labeled probe DNA was prepared using the Megaprime DNA Labeling System (Amersham Biosciences, Piscataway, NJ, USA) according to the manufacturer's instructions.

### Southern Blot Analysis

Southern blot analysis was performed according to the method described previously [31]. In brief, genomic DNA (20 µg) was subjected to gel-electrophoresis and transferred to a nylon membrane (Hybond N^+^, General Electric, New York, USA) with a few modifications. The membrane was hybridized with the [α-^32^P]dCTP-labeled cDNA probe of the *GUS* (1.8 kbp) fragment. Then the membranes were analyzed using a Bio Image Analyzer (BAS-2000, Fuji Photofilm, Tokyo, Japan) as described previously [32].

### Northern Blot Analysis

Total RNA was extracted from the young leaves of plants using the TRI reagents kit according to the manufacturer’s instructions. The total RNA gel blot analysis for transcript was performed as described previously [32] and the membrane was hybridized with the [α-^32^P]dCTP-labeled cDNA probes prepared from the pBI221, pTMVOM-L, pL11A-2-35 or pTW62-2 as mentioned above.

### siRNA Detection

Small RNA was prepared as described previously [32]. The enriched small RNA (30 μg) was separated by electrophoresis and electrotransferred to a nylon membrane. The probe was prepared as described above. Hybridization and siRNA detection was carried out with the labeled cDNA probes (*GUS*, *NtTOM1* or *NtTOM3*) as described previously [32].

### Evaluation of Virus Resistance

Purified ToMV-L [33], WMoV (TMV-W [34]) or TMV-OM [35] was used as inoculum. Detached 5 or 6th true leaves were cut out from the top of the scion and the base of the rootstock, respectively, and inoculated with a suspension of virus (10 μg/ml in 10 mM sodium phosphate buffer, pH 7.0). Detection of virus infection was performed 15 days after inoculation using ELISA and northern blot analysis. ELISA was performed using antiserum specific for each tobamovirus (Japan Plant Protection Association, Tokyo, Japan) and anti-rabbit IgG conjugated with alkaline phosphatase (Sigma-Aldrich, St. Louis, MO, USA).

## Results and Discussion

### Characterization of Sd1 Line Silenced with Both *NtTOM1* and *NtTOM3*

**Figure 1:**
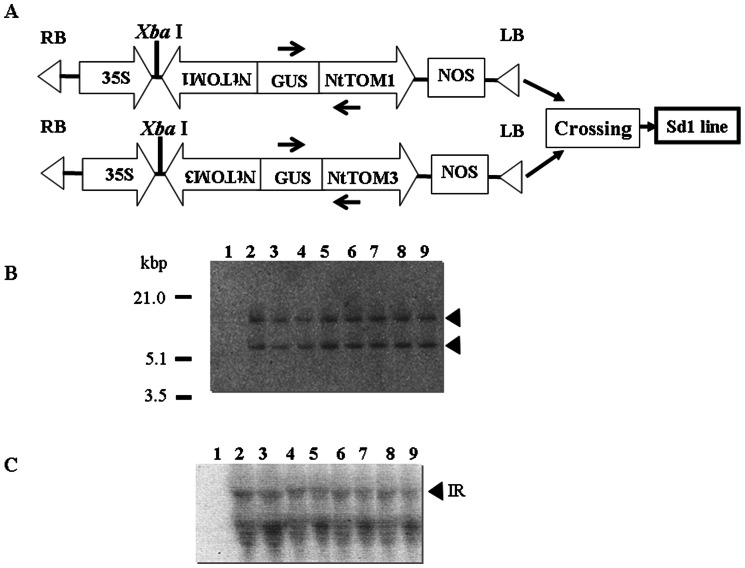
Southern and northern blot analyses of Sd1 line. (A) Schematic diagrams of the plasmid constructs used to produce Sd1 line [17]. (B) Southern blot analysis of transgenic Sd1 line with *GUS* probe. Genomic DNA (20 µg) was digested with *Xba* I and hybridized with the *GUS* probe labeled with [α-^32^P]dCTP. Lane 1, non-transgenic control plant; lanes 2–9, Sd1 line; black arrowheads represent positive signals. (C) Northern blot analysis of Sd1 line with *GUS* probe. Total RNA was hybridized with the [α-^32^P]dCTP-labeled cDNA probe prepared from the *GUS* fragment of pBI221. Lane 1, non-transgenic control plant; lanes 2–9, silenced Sd1 plants; IR, inverted repeat. Horizontal arrows show the positions of primers for PCR analysis.

The seeds of the crossed line between S-tom1 and S-tom3 were grown in the presence of kanamycin. All plants appeared normal in morphology and development. From the resulting collection of the progeny, all the plants of one Sd1 line showed two DNA bands on Southern blots probed by the GUS fragment (Figure 1B). On the other hand, another line showed only a single DNA band (Figure S2A). PCR analysis showed that the plants of that line with only the upper DNA band on the Southern blot carry the *NtTOM3*-IR transgene (Figure S2B). The total RNA from the plants of Sd1 was used for northern blot analyses. As a result, transgene expression was observed (Figure 1C) coinciding with the result reported previously [17].

### Graft Transmission of RNA Silencing from Sd1 line to Non-transgenic Tobacco Plants

**Figure 2:**
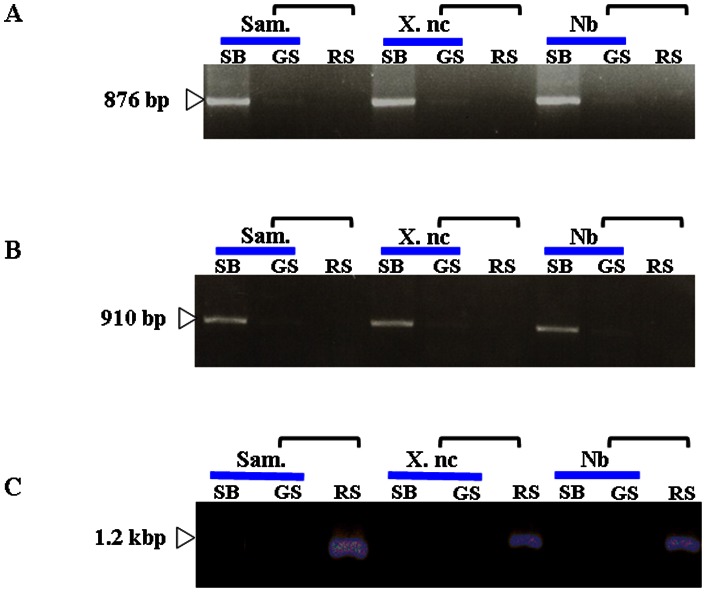
RT-PCR analysis of *NtTOM1* and *NtTOM3* in grafted plants. One microgram of total RNA was reverse-transcribed with NtTOM1-R, NtTOM3-R or GUS-linker-R primer. The cDNA was amplified by PCR under the conditions mentioned in Materials and Methods. The forward primer NtTOM1-F or NtTOM3-F and the reverse primer NtTOM1-R or NtTOM3-R were used to amplify the *NtTOM1 or NtTOM3* endogenous gene, respectively. *NtTOM3* transgene transcript was amplified with NtTOM3-F and GUS-linker-R. PCR products were analyzed by electrophoresis on a 1% agarose gel. SB, scion (before grafting); RS, rootstock; GS, grafted scion (after grafting). Horizontal bracket represents a grafted plant.

**Figure 3:**
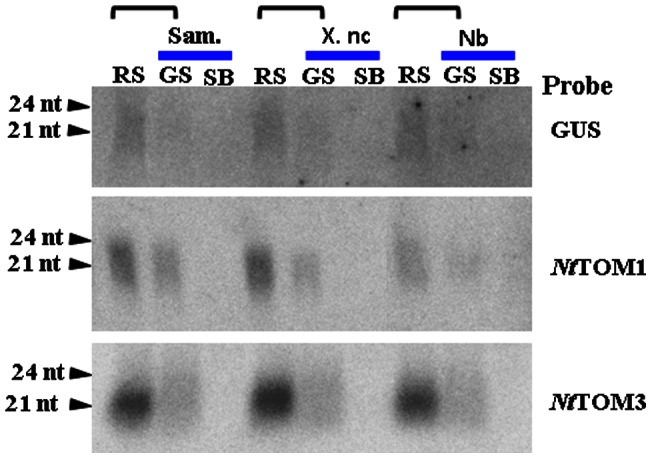
siRNA detection in grafted plants. Small RNA fractions (50 μg) were analyzed by northern hybridization with the [α-^32^P]dCTP-labeled cDNA probe prepared from *GUS*, *NtTOM1* or *NtTOM3.* SB, scion (before grafting); RS, rootstock; GS, grafted scion (after grafting). Horizontal bracket represents a grafted plant.

Non-transgenic scions of the same or different species of tobacco, *N. tabacum* L. cvs. Samsun and Xanthi nc or *N. benthamiana*, were grafted onto transgenic silenced Sd1 rootstock. Eight weeks after grafting, total RNA was extracted from leaves. Endogenous mRNA expression of both genes were highly detected in the non-transgenic scions before grafting but reduced extremely after grafting onto the Sd1 rootstocks, where it was under the detection level, (Figure 2A B). Furthermore, *NtTOM3* transgene transcript was only detected in rootstocks but not in scions (Figure 2C). The same result was also obtained with *NtTOM1* transgene (data not shown). The transgene derived siRNA was detected in both rootstocks and grafted scions (Figure 3). The RNA blots were re-hybridized with other probes specific to *NtTOM1* or *NtTOM3*. Using either probe, the corresponding siRNA was detected (Figure 3) even though the level was lower in scions than in rootstocks. Approximately 50–60% non-transgenic scions of tobacco showed the induction of RNA silencing (Table 1). From these results, it was confirmed that RNA silencing was induced in the non-transgenic grafted scions of not only the same species but also different species of tobacco.

**Table 1:** Induction of RNA silencing in non-transgenic scions.

Scions used on transgenic Sd1 rootstock	Number of plants used for grafting	Number of successful grafted plants	Number of tested plants	Number of silenced plants
Samsun	25	15	10	5
Xanthi nc	25	17	10	5
*N. benthamiana*	20	6	5	3

### Virus Resistance Assay of Scions after Grafting

**Figure 4:**
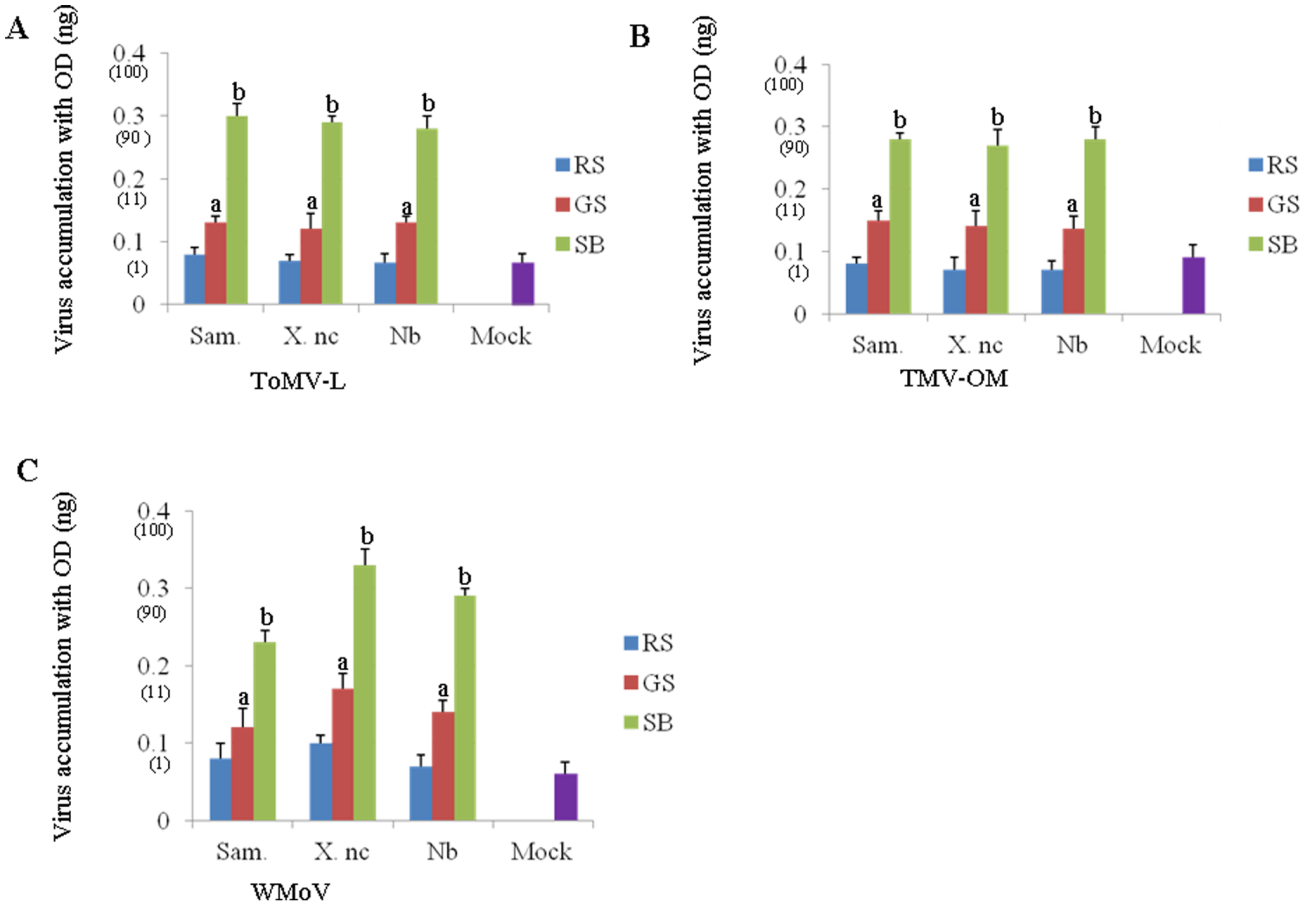
Virus resistance against tobamoviruses in both scions and rootstocks. (A) Purified ToMV-L [33], (B) TMV-OM [35] and (C) WMoV [34] were used as inoculum. Detached leaves were inoculated with a suspension of virus (10 μg/ml). Detection of viruses was performed 15 assays. The mean absorbance values with SD and different letter is significantly different from the mock days after inoculation using ELISA. Each absorbance value was analyzed in three independent ELISA (*p<0.05). SB, scion (before grafting); RS, rootstock; GS, grafted scion (after grafting).

**Figure 5:**
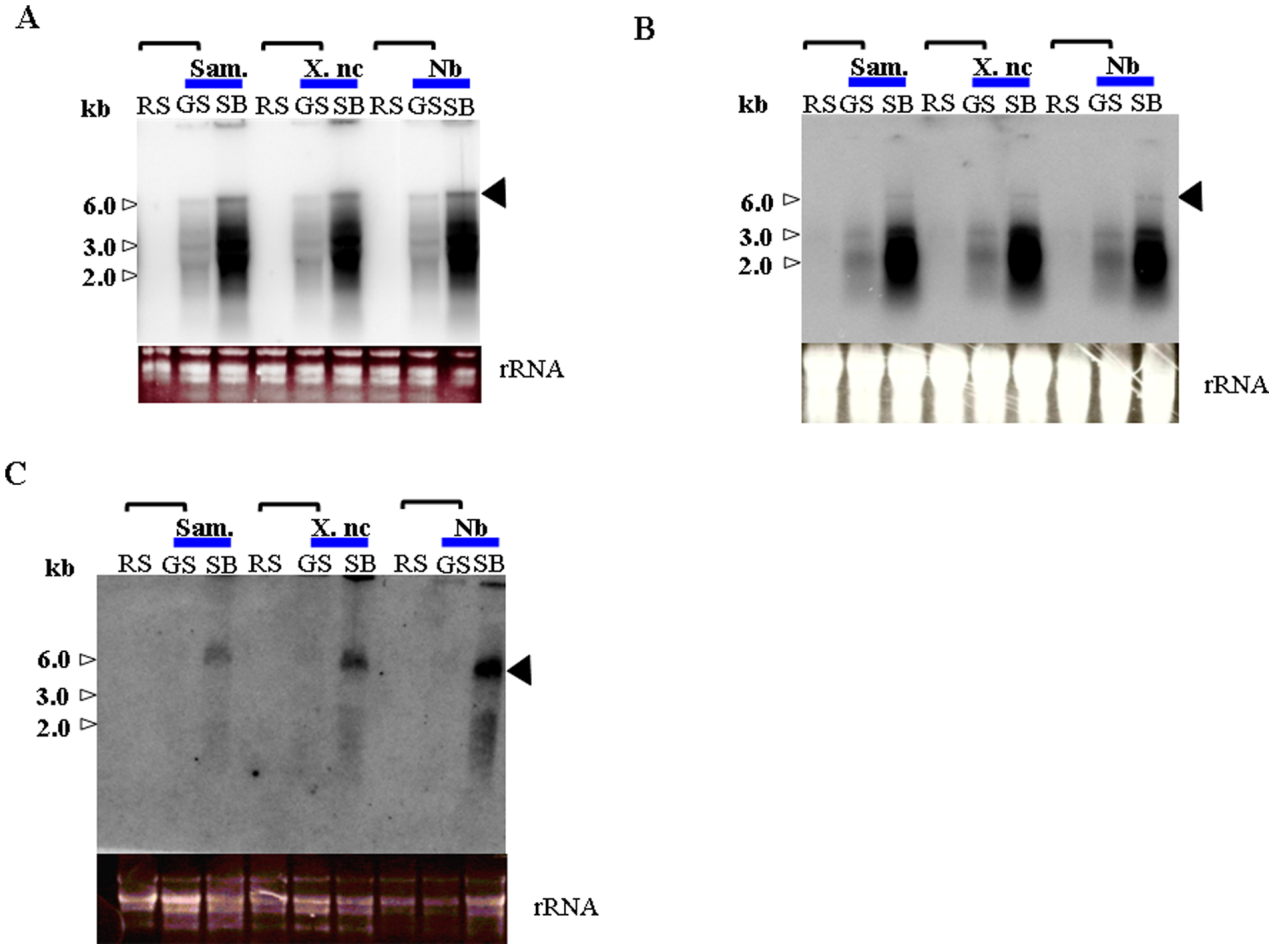
Northern blot analysis of virus inoculated leaves of grafted plants. Detached leaves were inoculated with a suspension of tobamovirus, ToMV-L, TMV-OM or WMoV at a concentration of 10 μg/ml. Northern blot analysis was performed 15 days after inoculation for detection of (A) ToMV-L, (B) TMV-OM or (C) WMoV. The [α-^32^P]dCTP-labeled cDNA probe was prepared as mentioned in Materials and Methods. Horizontal bracket represents a grafted plant.

It has been reported that the Sd1 lines show strong resistance to tobamoviruses [17]. Thus we examined whether any virus resistance was induced in the grafted scions. The leaves were detached from the scions 8 weeks after grafting and inoculated with several tobamoviruses including TMV-OM, ToMV-L and WMoV. Then virus accumulation was tested 16 days after inoculation by ELISA and northern blot analysis. As a result, an extremely low amount of virus was detected in grafted scions (Figure 4 and 5) showing that virus resistance was conferred. The non-transgenic scions grafted onto the non-transgenic rootstocks showed a high amount of virus accumulation similar to that in the non-transgenic scions before grafting onto the Sd1 rootstocks (Figure S3). *Cucumber mosaic virus* could multiply efficiently in both the non-transgenic scions and transgenic silenced rootstocks suggesting no resistance to other virus group than tobamovirus (unpublished data). This observation confirmed that non-transgenic scions were post-transcriptionally silenced after grafting onto the transgenically silenced rootstocks.

### Effect of Temperature on siRNA Accumulation and Virus Resistance

RNA silencing is negatively affected by low temperature [36]. Thus we examined the induction of RNA silencing in the scions of grafted plants at 24 and 15°C. After the grafted plants were kept at either 24 or 15°C for two months, siRNA accumulation was tested in both scions and rootstocks. There was no significant decrease in siRNA accumulation at low temperature (15°C) compared to that at 24°C (data not shown). The ToMV-L inoculation test showed that all scions and rootstocks were resistant to the virus at different temperatures (Figure S3). Moreover, almost similar levels of virus resistance were found at different temperatures in *N. tabacum* cv. Samsun and Xanthi nc although little difference, if any, in virus titer was found in *N. benthamiana*. These findings indicate that the resistance level is not remarkably different and rather similar between 24 and 15°C.

In this study, we showed the graft transmission of RNA silencing to non-transgenic scions from the silenced transgenic rootstocks where endogenous tobacco genes, *NtTOM1* and *NtTOM3*, were silenced by the hp of these genes driven by the 35S promoter. In micrografting experiments using *N. benthamiana,* it was reported that RNA silencing of the endogenous GSA gene is graft-transmissible to non-transgenic scions after grafting onto the transgenic rootstocks with hp of GSA gene driven by a companion cell-specific promoter [26]. However, it is not the case with transgenic rootstocks with the same hp GSA driven by the 35S promoter. This finding contradicts our results, which may be because their grafting duration was only for 2 weeks while ours was 8 weeks. Thus the prolonged duration may cause the successful graft transmission of RNA silencing in non-transgenic scions through grafting. In apple graft transmission of RNA silencing to a transgenic *GUS* expressing scions occurred in an *in vitro* system from *GUS* silenced transgenic rootstocks while the transmission to non-transgenic scions of high anthocyan natural variety did not from the *Mdans* silenced transgenic rootstocks [27]. It is not known why RNA silencing of the endogenous gene was not induced in the non-transgenic apple scions. It may be because the levels of siRNA in rootstocks and of *Mdans* transcript might have been insufficient to induce silencing in the non-transgenic scions. From our results, it is clear that RNA silencing can be induced in non-transgenic scions after grafting onto the transgenic silenced rootstocks.

Induction of RNA silencing in scions of different species, *N. benthamiana*, was also observed (Figure 3 and Table 1). Considering 97.2% identity of the nucleotide sequence between *N. tabacum* l. cv. Samsun (*NtTOM1*, AB193039 [17]) and *N. benthamiana* (*TOM1* homologue, AM261863 [37]), it is reasonable to suggest that RNA silencing was induced in the scion of *N. benthamiana*. It was shown that a transgenic silenced line of *N. benthamiana* showed resistance to TMV [37]. This approach may be applicable to other species of tobacco or crops of interest if the nucleotide sequence identity is high enough to induce RNA silencing.

Based on siRNA analysis and virus resistance assay against tobamoviruses, it was confirmed that the engineered transgene sequence in the Sd1 line as well as non-transgenic grafted scions was post-transcriptionally silenced. We analyzed the Sd1 rootstocks by Southern and northern blot analyses. It revealed that Sd1 plants carried one copy of each transgene and the expression of the transgenes was detected in the rootstocks (Figure 1B and C). The non-silenced non-transgenic scions grafted onto the silenced rootstocks showed accumulation of *NtTOM1* or *NtTOM3* siRNA and resistance to the tobamoviruses (Figure 3, 4, 5 and Table 1). These results suggest that the silencing signal which triggers silencing is transmitted from the silenced rootstocks to the non-transgenic scions and induced silencing.

Low temperature inhibited the accumulation of siRNA in insect, plant and mammalian cells [36], [38]–[40] and leads to enhancement of virus susceptibility and to a dramatic reduction in the level of siRNA [36]. However, another report showed that in *N. tabacum* no obvious decrease in siRNA accumulation was found at 15°C compared with that at 24°C [40]. In this study, we found no significant decrease in the accumulation of siRNA at low temperature in *N. tabacum* compared to that at 24°C. There was little or no reduction in the level of siRNA in *N. benthamiana* (15°C) (data not shown) in contrast to the remarkable reduction in siRNA accumulation [36]. However, in *N. tabacum*, there is no significant difference between 24 and 15°C [40]. Virus resistance assays showed that all scions were resistant to ToMV-L(Figure S3). Only a slight difference in virus titer by ELISA was found in the scions of *N. benthamiana* between the two temperatures. Thus effect of temperature on RNA silencing may depend upon the plant species, which might be due to the properties of silencing components in each plant species. These findings indicate that the resistance level might be similar at both 24°C and 15°C in this system.

In conclusion, RNA silencing can be induced in non-transgenic scions by grafting onto transgenic silenced rootstocks. This approach of grafting with non-transgenic scions onto transgenic rootstocks could be very useful for any crop, where grafting has been frequently used to confer desired traits. Grafting is conventionally used for vegetable and horticultural crops including tomato, cucurbitaceous plants and fruit trees. Their rootstocks are very highly resistant to soil-born disease pathogens and nematodes. Our findings may open the way to apply RNA silencing to crops for conferring other desired traits such as higher nutritional value, improved flavor, and sugar contents.

## Supporting Information

Figure S1 **Analysis of one progeny line crossed by S-tom1 and S-tom3.** (A) Southern blot analysis of progeny line with *GUS* probe. Genomic DNA was digested with *Xba* I and hybridized with the *GUS* probe labeled with [α-^32^P]dCTP. Each lane represents the genomic DNA of a progeny line. Lane 1, non-transgenic control plant; lanes 2–10, one progeny line plants; lane 11, Sd1; black arrowhead represents positive signal. (B) PCR analysis for the presence of the transgene using GUS-linker-F and NtTOM3-R primers. Lane 1, Sd1; lanes 2–8, T2 plants; lane 9, non-transgenic control. (C) PCR analysis for the presence of the transgene using GUS-linker-F and NtTOM1-R primers. Lane 1, Sd1; lanes 2–8, T2 plants; lane 9, non-transgenic control. DNA (100 ng) was used as template for PCR. Black arrow represents PCR amplified DNA.
(TIF)

Figure S2 **Grafted plants.** Photographs were taken eight weeks after grafting. Transgenic silenced Sd1 line used as rootstocks and non-transgenic scions; (A), *Nicotiana tabacum* cv. Samsun; (B), *N. tabacum* cv. Xanthi nc and (C), *N. benthamiana*.
(TIF)

Figure S3 **Virus resistance assay in control grafted plants.** (A) Purified ToMV-L was used as inoculum. Detached leaves were inoculated with a suspension of virus (10 μg/ml). Detection of viruses was performed 15 days after inoculation using ELISA as shown in Figure 4. (B) Northern blot analysis was performed 15 days after inoculation for detection of ToMV-L. The [α-^32^P]dCTP-labeled cDNA probe was prepared as mentioned in Materials and Methods. SB, scion (before grafting); RS, rootstock of Sd1; GS, grafted scion (after grafting), CGS, grafted scion on control rootstock. Horizontal bracket represents a grafted plant.
(TIF)

Figure S4 **Effect of temperature on virus resistance in the grafted scions.** Purified ToMV-L [33] was used as inoculum. Detached leaves were inoculated with a suspension of virus (10 μg/ml). Detection of viruses was performed 15 days after inoculation using ELISA. The mean absorbance values are shown with SD. Each absorbance value was analyzed in three independent ELISA assays. Asterisks indicate significant difference from 24°C (*p<0.01).
(TIF)

Table S1 List of primers used in this study.
(DOC)
