# Supplementary figures and images for: Graft Transmission of RNA Silencing to Non-Transgenic Scions for Conferring Virus Resistance in Tobacco
Source: PLoS One. 2013 May 22;8(5):e63257. doi: 10.1371/journal.pone.0063257 (PMC3661558; doi:10.1371/journal.pone.0063257)

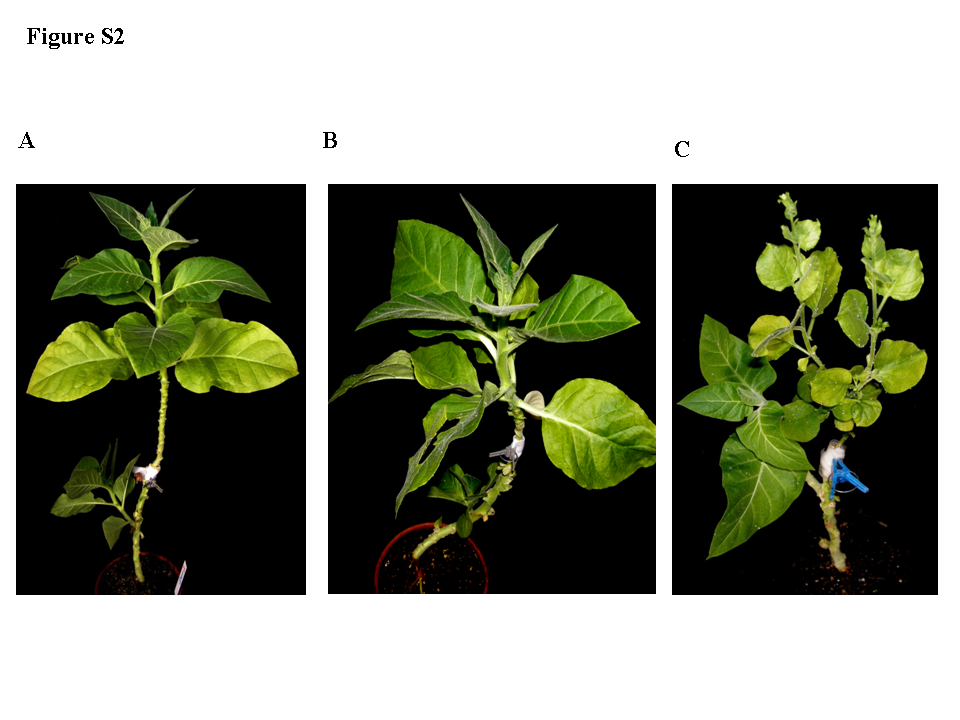

Supplement: Figure S2 — Grafted plants. Photographs were taken eight weeks after grafting. Transgenic silenced Sd1 line used as rootstocks and non-transgenic scions; (A), Nicotiana tabacum cv. Samsun; (B), N. tabacum cv. Xanthi nc and (C), N. benthamiana. (TIF) [file pone.0063257.s002.tif]

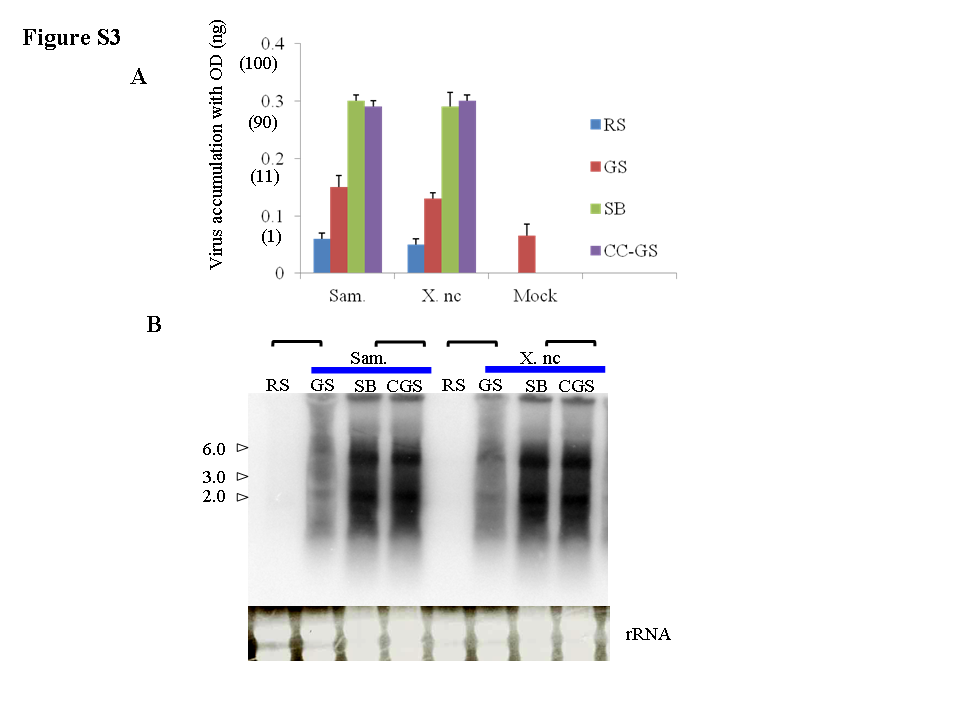

Supplement: Figure S3 — Virus resistance assay in control grafted plants. (A) Purified ToMV-L was used as inoculum. Detached leaves were inoculated with a suspension of virus (10 μg/ml). Detection of viruses was performed 15 days after inoculation using ELISA as shown in Figure 4. (B) Northern blot analysis was performed 15 days after inoculation for detection of ToMV-L. The [α-32P]dCTP-labeled cDNA probe was prepared as mentioned in Materials and Methods. SB, scion (before grafting); RS, rootstock of Sd1; GS, grafted scion (after grafting), CGS, grafted scion on control rootstock. Horizontal bracket represents a grafted plant. (TIF) [file pone.0063257.s003.tif]

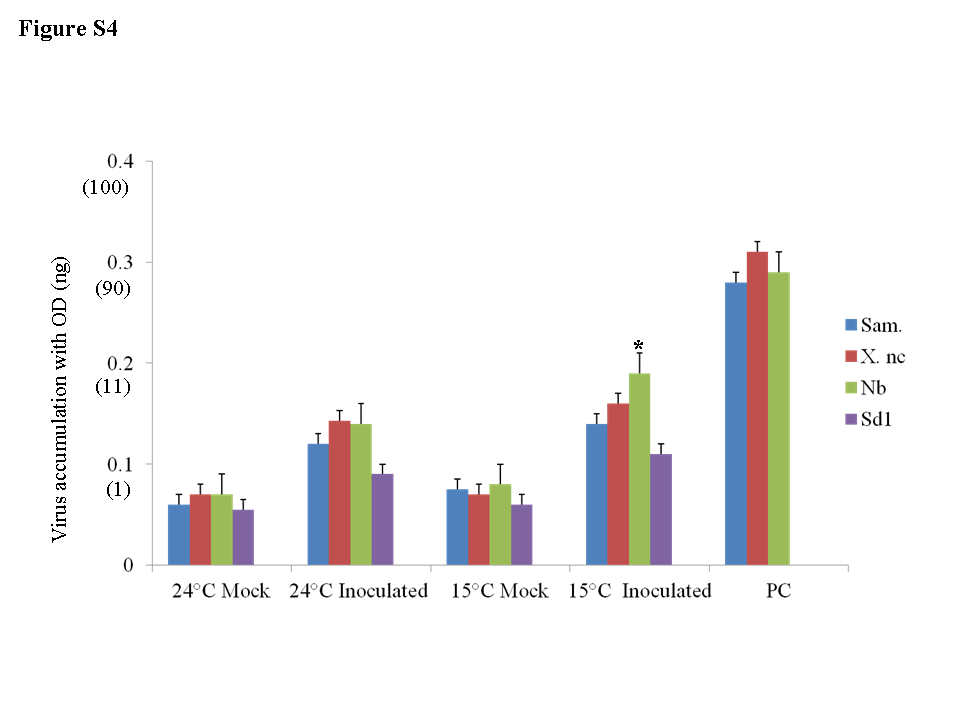

Supplement: Figure S4 — Effect of temperature on virus resistance in the grafted scions. Purified ToMV-L [33] was used as inoculum. Detached leaves were inoculated with a suspension of virus (10 μg/ml). Detection of viruses was performed 15 days after inoculation using ELISA. The mean absorbance values are shown with SD. Each absorbance value was analyzed in three independent ELISA assays. Asterisks indicate significant difference from 24°C (*p<0.01). (TIF) [file pone.0063257.s004.tif]
