# Supplementary material for: Graft Transmission of RNA Silencing to Non-Transgenic Scions for Conferring Virus Resistance in Tobacco
Source: PLoS One. 2013 May 22;8(5):e63257. doi: 10.1371/journal.pone.0063257 (PMC3661558; doi:10.1371/journal.pone.0063257)
Supplement: Table S1 — List of primers used in this study. (DOC) [file pone.0063257.s005.doc]

**Supporting Information**

**Table S1 List of primers used in this study.**

| Primer name | Nucleotide sequence |
| --- | --- |
| GUS-linker-F | 5′-CATGAAGATGCGGACTTACG-3′ |
| GUS-linker-R  NtTOM1-F  NtTOM1-R  NtTOM3-F  NtTOM3-R | 5′-GGCTTATGCCGCACCTA-3′  5′-ACAGGCCAACCAATTTAAAGGACAG-3′  5′-TCGCAGGATGTATAGCACAAGT-3′  5′-TGATTTGAATTTTGGAATCTCCGGC-3′  5′-TAGCGAATAGGGTGGTACTGTGT-3′ |
